# Psychometric properties of the risk, pain, and injury questionnaire in Chinese collegiate athletes and its relationship with locus of control

**DOI:** 10.1371/journal.pone.0281011

**Published:** 2023-01-27

**Authors:** Zelei Liu, Hongbo Zhao, Yuqiong Cai, Long Sun

**Affiliations:** 1 School of Physical Education, Liaoning Normal University, Dalian, Liaoning, P. R. China; 2 School of Psychology, Liaoning Normal University, Dalian, Liaoning, P. R. China; 3 Physical Education Section, Dalian No.24 High School, Dalian, Liaoning, P. R. China; Lorestan University, ISLAMIC REPUBLIC OF IRAN

## Abstract

**Purpose:**

This study aimed to adapt the Risk, Pain, and Injury Questionnaire (RPIQ) to Chinese collegiate athletes and examine its reliability and validity.

**Methods:**

Six hundred thirty collegiate athletes aged 17 to 24 years agreed to complete the RPIQ, the Chinese version of the SIAS and an LOC scale. Exploratory factor analysis (EFA, *n* = 300) and confirmatory factor analysis (CFA, *n* = 330) were conducted to explore its structure, and convergent and construct validity were investigated by examining the relationships between various factors of the RPIQ, LOC, SIAS and injury risk.

**Results:**

The results of EFA show the Chinese version of the RPIQ contained 12 items and was divided into three factors: tough, pressed and rational choice. CFA confirmed its factorial structure (*CMIN/DF* = 2.61, *CFI* = 0.93, *TLI* = 0.91, *RMSEA* = 0.07). The reliability of the scale was satisfactory (Cronbach’s alpha = 0.75). Significant associations between the RPIQ factors and LOC and SIAS were found, suggesting the construct validity of the scale was acceptable. Additionally, significant gender differences were found in the RPIQ factors and athletes who participated in individual sports scored higher on tough and rational choice factors than those who participated in team sports.

**Conclusions:**

The Chinese version of the RPIQ has sufficient psychometric properties and can be used as a reliable and effective tool for measuring attitudes of the risk, pain and injury of collegiate athletes.

## 1 Introduction

With the development of the worldwide economy and the improvement of living standards, an increasing number of people participate in various sports. However, there is a high risk that many people will become injured upon participating in sports. Statistics show that approximately 750,000 college students in the United States develop sports-related injuries each year [1]. According to a survey conducted in 2015, the rate of sports injury in China has been rising similarly, with an average annual increase of more than 45% [2]. However, due to the lack of a valid instrument to assess the attitudes of Chinese collegiate athletes to risk, pain and injury, the present study aimed to develop a questionnaire and to examine its reliability and validity.

Sports injuries have been the focus of studies for decades. As early as 1983, researchers began to pay attention to sports injuries and studied the subcultural dimension of how professional athletes play with pain and talk about injury [3]. In 1992, researchers developed the Exercise Pain Scale (SIP), which consists of 25 entries (e.g., Do anything to get my mind off the pain [4]. In 1996, Nixon developed the Risk, Pain, and Injury Questionnaire (RPIQ) [5–7]. In 2006, researchers developed the Sports Injury Anxiety Scale (SIAS) using American collegiate athletes, which contained 29 items [8]. The reliability and validity of the scales were satisfactory and they can be used to measure athletes’ attitudes toward competition after facing injury risk, pain and injury.

### 1.1 Risk, pain, and injury questionnaire

The RPIQ consists of 31 items that can be divided into three factors: tough, pressed and rational choice. Tough refers to the ability to show resilience in the face of risk, pain and injury, pressed refers to continuing to play in the face of pressure from coaches and spectators after injury, and rational choice refers to an athlete’s willingness to accept the risk of an exercise [9]. The RPIQ has shown satisfactory reliability in previous studies and has broadened the understanding of the willingness of athletes to continue to participate in sports after injury [6, 7].

The construct validity of the RPIQ was further examined in 2005. Walk and Wiersma re-examined the model fit of the original RPIQ and found the results were not satisfactory [10]. Correspondingly, a 13-item questionnaire was developed with improved reliability and validity [10]. The reliability of the 13-item questionnaire among collegiate athletes remained satisfactory [11]. For example, some studies have found that athletes with a stronger athletic identity show a more positive attitude when competing in pain [11]. All these studies provided evidence for the usefulness of the RPIQ in assessing collegiate students’ sports risk, pain and injury.

Due to cultural differences, the RPIQ needed to be restructured in the context of Chinese culture and language before it could be used to assess risk, pain and injury in Chinese athletes. Prior studies have demonstrated that Chinese and American athletes differentiate in internal motivations, confidence, motivation and overall mental skills when participating in sports. These differences may be underlying causes shaping the different attitudes toward risk, pain and injuries [12]. Chinese athletes also scored higher than American athletes in terms of confidence, motivation and overall mental skills [13]. These cultural differences highlight the need to adapt the original RPIQ into Chinese to develop a culturally sensitive instrument. To the best of our knowledge, the relevant studies regarding attitudes of Chinese collegiate athletes to risk, pain and injury are very limited.

### 1.2 Athletes’ risk, pain and injury and locus of control

Among the factors that affect athletes’ risk, pain and injury scores, LOC is an important factor [14–17]. Locus of control (LOC) refers to personality attributes reflecting the degree to which a person generally perceives events to be under their own control (internal LOC) or under the control of others or other outside forces (external LOC) [17]. LOC influences individuals’ behaviors in many aspects, such as sports psychology [14–17], traffic psychology [18, 19] and health psychology [20]. In sports-relevant studies, researchers have found that participants in individual sports are more likely to believe that the results of the sport can be attributed to their own efforts [21]. Another study showed that Chinese people are more dependent on others, while Americans prefer to deal with things independently [16]. Hence, American athletes are more likely to continue competing in individual events, while Chinese athletes are more likely to continue competing in team events [22]. Given the relationship between the LOC and athletes’ attitude towards risk, pain and injury, the LOC can be used to validate the RPIQ in this study.

### 1.3 Athletes’ risk, pain and injury and demographic factors

Regarding the associations between demographic factors and RPIQ factors, studies have shown controversial results. A study found that women tend to be more anxious and nervous in the face of risk, pain and injury and usually adopt emotion-centered coping strategies, while men are generally more rational [21]. Other studies found no gender differences in athletes’ attitude towards risk, pain and injury [11, 23]. Regarding training experience, only one study found that the number of sports injuries increases with years of training [9]. However, considering the differences between Chinese and American college athletes, this study will further explore the relationship between demographic factors and the various RPIQ factors [24].

### 1.4 Athletes’ risk, pain and injury, injury risk and sport type

Furthermore, the RPIQ factors are closely related to injury risk and are affected by sport type. Studies have shown that athletes engaged in high-risk activities tend to be calmer, more rational and better able to control their emotions [25, 26]. Athletes who compete in team sports have a harder time experiencing risk, pain and injury than those who compete in individual sports. As a member of a team, athletes work together in harmony, which may be due to a strong collective sense of honor; as a result, athletes usually choose to carry on playing even if they experience risk, pain and injury [27]. However, athletes in individual sports had a stronger willingness to win as compared to those in team sports. Consequently, they devote more effort to the pursuit of achievement and persist in finishing even if they experience risk, pain and injury [28]. Therefore, the associations between RPIQ factors, sport types and injury risk will be further examined to validate the RPIQ in Chinese collegiate athletes.

### 1.5 Aim of the present study

The main purpose of the present study is to adapt the 13-item RPIQ [10] to Chinese language and culture. We expect that the factorial structure of this scale will be similar to that of original scale, and its reliability is acceptable. To achieve this goal, exploratory factor analysis (EFA) and confirmatory factor analysis (CFA) were conducted to explore its structure. The convergent and construct validity of the scale were investigated by examining the relationships between the factors of the RPIQ, SIAS, LOC and injury risk. We expected that the factors of the RPIQ are negatively correlated with LOC and positively correlated with SIAS, and collegiate athletes who participated in low-risk sports scored higher on tough and rational choice factors than those who participated in high-risk sports.

## 2 Method

### 2.1 Participants

The present study was approved by the Logistics Department for Civilian Ethics Committee of Liaoning Normal University. The participants were sampled using a "snowball" technique: questionnaires were distributed to an initial sample of college students, who had friends, classmates and acquaintances with athletic experiences complete them [29]. Six hundred fifty athletes aged between 17 and 24 were recruited from Beijing Sport University, Shanghai University of Sport, Liaoning Normal University and Shenyang Sport University and they agreed to participate in this study. These universities are represented in the field of sports in China. The inclusion criteria were a) to have the professional training for at least one year and, b) to be a college athlete. Overall, six hundred and thirty (96.92% response rate) questionnaires were returned. The data of 20 participants were discarded due to missing responses.

The final sample included 471 males (74.76%) and 159 females (25.24%). Most of the participants were students majoring in physical education, and some of them had participated in national and provincial competitions. The participants competed in cricket (*n* = 3), bludgeon (*n* = 4), baseball (*n* = 7), Chinese winter sports (*n* = 9), rugby (*n* = 24), golf (*n* = 4), boxing (*n* = 5), bicycle (*n* = 2), fencing (*n* = 2), fitness (*n* = 12), basketball (*n* = 107), softball (*n* = 8), roller skating (*n* = 3), volleyball (*n* = 21), rock climbing (*n* = 1), kayaking (*n* = 2), table tennis (*n* = 22), hockey (*n* = 3), shooting (*n* = 2), handball (*n* = 5), wrestling (*n* = 3), taekwondo(*n* = 3), gymnastics (*n* = 4), sports dance (*n* = 17), track and field (*n* = 168), tennis (*n* = 12), martial arts (*n* = 61), swimming (*n* = 15), badminton (*n* = 31), football (*n* = 70). A total of 263 athletes (41.75%) participated in team sports. Team sports included cricket, bludgeon, baseball, rugby, basketball, softball, volleyball, kayaking, hockey, handball and football. A total of 367 athletes (58.25%) participated in individual sports. Individual sports included table tennis, Chinese winter sports, golf, boxing, fencing, fitness, roller skating, shooting, rock climbing, wrestling, taekwondo, gymnastics, sport dance, track and field, tennis, martial arts, swimming, badminton and bicycle.

The sample was divided into two sub-samples according to the date of data collection. The first sample was collected from September 25, 2020 to October 12, 2020 and included 300 participants. The second sample was collected from November 20, 2020 to December 10, 2020 and included 330 participants. The participants in sample 1 were all student athletes aged 18–24 years. There were 195 males and 105 females. Participants had 0.5–8 years of athletic competition experience. The number of injuries in college students ranged from 0 to 25. The first sample was used for exploratory factor analysis.

The participants in sample 2 were all student athletes aged 17 to 24 years. There were 276 males and 54 females. Participants had between 0.5 and 12 years of athletic competition experience. The number of injuries experienced by college students ranged from 0 to 25. Table 1 shows detailed demographic information. The second sample was used for confirmatory factor analysis.

**Table 1:** Demographic information of participants.

Demographic variables	Sample 1 (*n* = 300)				Sample 2 (*n* = 330)			
	Min	Max	Mean	SD	Min	Max	Mean	SD
Age (year)	18	24	20.84	1.67	17	24	20.45	1.36
Gender ^a^	1	2	1.35	0.48	1	2	1.16	0.37
Years of training	0.5	8	4.20	2.06	0.5	12	5.47	2.84
Number of injuries	0	25	3.76	4.49	0	25	5.34	6.22

^a^ 1 = Male, ^a^ 2 = Female; Mean was reported.

*p < 0.05

**p < 0.01

### 2.2 Material

#### 2.2.1 Risk, pain, and injury questionnaire

The original RPIQ consisted of 13 items and was divided into three factors [11]. The three factors are "tough" (4 items), "pressed" (4 items) and "rational choice" (5 items). Participants were asked to rate each question on a 4-point Likert questionnaire ranging from 1 (strongly agree) to 4 (strongly disagree).

The original RPIQ was first translated into Chinese following the translation/ back-translation procedure. The RPIQ was translated by an independent Chinese researcher, and this version was subsequently translated into English by a bilingual Chinese and English researcher. Two sports injury specialists (1 female, average age 43, mainly engaged in sports injury and sports psychology related fields) were invited to read and evaluate the accuracy of the items. The translated questionnaire was initially distributed to twenty people and asked them to check whether there were any ambiguities or relevant expressions needed to be modified. We compared the differences between the translated questionnaire and the original questionnaire, and some minor revisions were made correspondingly. After the differences in these translations were properly addressed, Higher level of agreement between the two experts (Inter-rater reliability 0.73–0.92) was got an initial 13-item questionnaire was obtained.

#### 2.2.2 Locus of control scale

Rotter’s Locus of Control scale (LOC) [18], was used in this study. This scale was translated and adapted to Chinese language by Wang [30]. The scale consisted of 23 items and 6 insertion items. Each item contained a statement of internal control and a statement of external control. Higher score indicates more external control tendency. The reliability and validity are acceptable in Chinese samples [30, 31]. Participants were asked to choose an answer for each item. The internal reliability of the scale was 0.59 in this study.

#### 2.2.3 Sports injury anxiety scale

The 28-item Chinese version of the Sport Injury Anxiety Scale (SIAS) measures injury anxiety in athletes [32]. The SIAS scale consists of six dimensions, including loss of athleticism (5 items), being perceived as weak (4 items), experiencing pain (6 items), loss of social support (4 items), reinjury (4 items), letting down important others (5 items). Participants rated each item on a 6-point Likert scale ranging from 1 (strongly disagree) to 6 (strongly agree). In this study, the internal consistency reliability of the SIAS was 0.69–0.83.

#### 2.2.4 Demographic questionnaire

Participants were also asked to provide demographic information, including gender, age, risk of injury, years of training and number of injuries during college.

### 2.3 Procedures

The verbal informed consent was obtained from all participants for inclusion in the study. The RPIQ-C, SIAS and LOC were administrated to 650 collegiate students at Beijing Sport University, Shanghai University of Sport, Liaoning Normal University and Shenyang Sports University, respectively. A pen and paper test was administrated to all the participants. Before the test, participants were informed of the test purpose of the study. After asking for the consent of the participants, participants signed the informed consent form and were required to complete the questionnaires within 30 minutes. Upon completion of the submission, each participant received a small gift (10 RMB).

### 2.4 Data analysis

The data were analyzed using SPSS 18.0. First, the statistical properties of the items were examined using sample 1, including the mean, SD, corrected item-total correction, factor loading and community. Second, an exploratory factor analysis (EFA) was conducted using sample 1 via SPSS18.0 to explore the factorial structure of the RPIQ. Reliability analysis was conducted to examine the internal consistency of the RPIQ factors, with a Cronbach’s alpha value greater than 0.6 expected [33]. Third, to assess the model fitness of the RPIQ, a confirmatory factor analysis (CFA) was conducted using sample 2 via AMOS 23.0. To test the concurrent validity and divergent validity, the Pearson correlation between RPIQ-C factor, LOC and SIAS were analyzed using sample 1. Finally, one-way ANOVAs were conducted using sample 1 to examine differences in RPIQ-C factors by gender, sport type, and risk of injury.

### 2.5 Multi colinearity check

We assessed common method bias using Harman’s single factor method [34]. The first factor accounted for 10.34% of the variance, and factors whose eigenvalues exceeded 1.0 accounted for 60.78% of the variance. Thus, common method bias does not appear to be a serious problem for this study.

## 3 Results

### 3.1 Descriptive statistics

The descriptive properties of the items were first analyzed. The mean, standard deviations, corrected item-total, Factor loading and Community are shown in Table 2. The mean of each item ranged from 1.8 to 3.0, showing that there were no ceiling or floor effects. The corrected item-total of each item were more than 0.3.

**Table 2:** Descriptive statistics of RPIQ items.

Items	Mean	SD	Corrected item-total	Factor loading	Community
**Tough**					
1 Athletes should “tough it out” with an injury or pain today and not worry about the effects tomorrow.	2.48	0.82	0.60**	0.83	0.73
2 Athletes should ignore the pain.	2.94	0.73	0.53**	0.65	0.63
3 Playing with injuries and pain demonstrates character and courage	2.13	0.94	0.61**	0.76	0.63
**Pressed**					
4 Coaches make athletes feel guilty if they do not want to play hurt or with pain	2.77	0.69	0.50**	0.70	0.57
5 Coaches only care about their players who are healthy and able to play.	2.75	0.75	0.48**	0.80	0.64
6 Coaches say they do not want athletes to play with serious injuries, but they actually push them to play if they are needed.	2.72	0.75	0.47**	0.83	0.69
7 Coaches are impressed with athletes who play with injuries and pain.	2.37	0.68	0.45**	0.47	0.41
**Rational choice**					
8 Athletes who endure pain and play hurt deserve our respect.	1.82	0.68	0.36**	0.72	0.57
9 Athletes who care about their team will try to play with injuries and pain.	2.08	0.66	0.53**	0.64	0.44
10 Every athlete should expect to have to play with an injury or pain sometime.	2.21	0.69	0.55**	0.68	0.49
11 Only athletes understand what it is like to play with injuries and pain.	2.04	0.66	0.53**	0.74	0.57
12 Athletes will do everything possible to play despite injuries and pain.	2.13	0.71	0.57**	0.69	0.52

*p < 0.05

**p < 0.01

### 3.2 Exploratory factor analysis

All 13 items were submitted to an EFA using principal component extraction with varimax rotation using the data of sample 1. The absolute value of the factor loadings is chosen as 0.40. Items with factor loadings below 0.40 are considered inadequate and are generally recommended for elimination. The results showed a three-factor structure that could explain 59.28% of the total variance (KMO = 0.843, Bartlett = 1116.41, *p* < 0.001). In line with the original questionnaire, the three factors were named tough, pressed, and rational choice. The factor loadings are shown in Table 2. One item (i.e., no pain, no gain) that loaded more than one factor was discarded. In the original RPIQ, this item belonged to the tough factor.

### 3.3 Confirmatory factor analysis

The model fit of the RPIQ-C was assessed by a confirmatory factor analysis via AMOS 23.0 using data from sample 2. The model fit of the original 13-item questionnaire was first examined. The results showed the model fit were not acceptable (*CMIN* = 214.94, *DF* = 62, *CMIN/DF* = 3.47, *CFI* = 0.87, *TLI* = 0.84, *RMSEA* = 0.087). The model fit of the 12-item structure was then examined. The model fit was improved this time (*CMIN* = 132.86, *DF* = 51, *CMIN/DF* = 2.61, *CFI* = 0.93, *TLI* = 0.91, *RMSEA* = 0.07). According to the criteria proposed by Hu and Bentler [33], the value of CFI and TLI greater than 0.9, RMSEA<0.08, indicates that the model fitness indices of RPIQ-C were acceptable. The path diagram is shown in S1 Fig.

### 3.4 Reliability of the RPIQ-C

Sample 1 was used to examine the reliability of the RPIQ-C. According to the criteria proposed by Powell and Dompier [35], the value of the Cronbach’s alpha should be at least greater than 0.7. Reliability analysis showed the internal consistency (Cronbach’s alpha) for tough factor was 0.70, 0.69 for pressed factor and 0.76 for rational choice factor.

### 3.5 Convergent and construct validity of the RPIQ-C

To test the concurrent validity and divergent validity, we used the sample 1 to assess the correlations between RPIQ factors, SIAS and LOC scales (see Table 3). The significant associations between the RPIQ-C factors, LOC scale and the SIAS factors showed the construct validity of the scale were acceptable.

**Table 3:** Correlations between the RPIQ-C factors, SIAS and LOC.

RPIQ-C factors	1 Tough	2 Pressed	3 Rational Choice
Tough	-		
Pressed	0.36**	-	
Rational Choice	0.24**	0.17**	-
Losing Athleticism	0.25**	0.22**	0.30**
Being Perceived as Weak	0.17**	0.05	0.21**
Experiencing Pain	0.36**	0.22**	0.14*
Loss of Social Support	0.17**	0.12*	0.09
Reinjury	0.30**	0.23**	0.10
Letting Down Important Others	0.08	0.05	0.18**
LOC	-0.43**	-0.25**	-0.29**

*p < 0.05

**p < 0.01

Table 3 shows that tough, pressed and rational choice factors were negatively correlated with LOC. Most factors of SIAS were positively with the RPIQ-C factors. Additionally, the three factors of the RPIQ-C were positively correlated with each other, indicating that the content validity of the scale was acceptable.

### 3.6 RPIQ-C factors and demographic factors

Univariance of MANCOVA was conducted with the factors of the RPIQ as dependent variables, the demographic variables as covariates. The results showed that the effects of gender, (*F*_[__3__, 294]_ = 4.55, *p* < 0.01, *η2 p* = 0.44), and years of training, (*F*_[__3__, 294]_ = 5.63, *p* < 0.01, *η2 p* = .005), were significant. The effect of age, (*F*_[__3__, 294]_ = 0.25, *p* > 0.05, *η2 p* = .002) was not significant. The mean and standard deviation for males and females in RPIQ-C factors are shown in Table 4.

**Table 4:** Differences in the RPIQ-C factors by gender.

RPIQ-C factors	Male (*n* = 195)		Female (*n* = 105)		*F* [2,297]
	*M*	*SD*	*M*	*SD*	
Tough	2.44	0.68	2.65	0.60	7.33**
Pressed	2.62	0.54	2.71	0.46	2.10
Rational Choice	2.00	0.50	2.17	0.44	8.70**

*p < 0.05

** *p* < 0.01

One-way ANOVA was conducted with the factors of the RPIQ as dependent variables, the gender as independent variable. The results showed that males scored significantly lower on tough and rational choice factors than females. No gender difference was found on pressed factor. Pearson correlations showed that the pressed factor was positively correlated with years of training (*r* = 0.17, *p* < 0.01).

### 3.7 Differences in the RPIQ-C factors by risk of injury and sport type

#### 3.7.1 Risk of injury

Participants were divided into three groups with low, medium and high risk of injury (see Table 5). Low-risk sports include swimming, tennis, track and field, kayaking and golf. Medium-risk sports include taekwondo, table tennis, baseball, martial arts, hockey, volleyball, softball, badminton, basketball, fitness, cricket, sports dance, and Chinese winter sports. High-risk sports include football, gymnastics, roller skating, boxing, rock climbing, and handball.

**Table 5:** Differences in the RPIQ-C factors by risk of injury.

RPIQ-C factors	Low-risk (*n* = 115)		Medium-risk (*n* = 149)		High-risk (*n* = 36)		*F* [2,297]
	*M*	*SD*	*M*	*SD*	*M*	*SD*	
Tough	2.57	0.70	2.50	0.62	2.44	0.66	0.66
Pressed	2.70	0.52	2.62	0.50	2.63	0.56	1.01
Rational Choice	2.20	0.49	2.00	0.44	2.00	0.59	3.55*

*p < 0.05

** *p* < 0.01

One-way ANOVA was conducted with the factors of the RPIQ as dependent variables, the risk of Injury as independent variable. The results showed that differences were found on rational choice factor among the three groups, (*F*_[__2__, 297]_ = 3.55, *p* < 0.05, *η*2 p = 0.023). Post-hoc tests revealed that athletes participating in low-risk sports scored higher than athletes participating in high-risk sports (*p* < 0.05).

#### 3.7.2 Sport type

The mean and standard deviation of individual and team sports in the RPIQ-C factors are shown in Table 6.

**Table 6:** Differences in the RPIQ-C factors by types of sports.

RPIQ-C factors	Individual Sports (*n* = 191)		Team sports (*n* = 109)		*F* [2,297]
	*M*	*SD*	*M*	*SD*	
Tough	2.58	0.68	2.39	0.62	5.92*
Pressed	2.67	0.50	2.63	0.55	0.39
Rational Choice	2.11	0.46	2.00	0.51	6.34*

*p < 0.05

** *p* < 0.01

One-way ANOVA was conducted with the factors of the RPIQ as dependent variables, the types of sports as independent variable. The results showed that there were differences in the Risk, Pain, and Injury Questionnaire related to tough, (*F*_[__1__, 298]_ = 5.92, *p* < 0.05, *η*2 p = 0.019) and rational choice, (*F*_[1, 298)_ = 6.34, *p* < 0.05, *η*2 p = 0.021). Athletes in individual sports scored higher on the tough and rational choice factors than those who participated in team sports.

## 4 Discussion

This study aimed to adapt the Risk, Pain, and Injury Questionnaire (RPIQ) to Chinese collegiate athletes and assess whether it can accurately assess Chinese athletes’ attitudes toward risk, pain and injury in sports. The findings showed that the reliability and validity of the Chinese version of the RPIQ are satisfactory.

First, the results of this study showed that the factorial structure of the RPIQ remained stable across cultures [10]. The final Chinese version of the RPIQ contained 12 items, including three dimensions: tough factor, pressed factor and rational choice factor. The factorial structure of the RPIQ was confirmed by confirmatory factor analysis. There were significant positive correlations between the three factors of the RPIQ, which was not reported in the original and revised questionnaire [6, 10]. Although one item was deleted, the reliability (ranging from 0.63 to 0.78) of the three factors was acceptable.

Second, the RPIQ-C was also significantly positively correlated with the LOC scale, supporting its construct validity, and was significantly positively associated with both “losing athleticism” and “loss of social support” factors of the SIAS, providing support for its convergent validity. More importantly, LOC was an effective predictor of RPIQ-C factors after controlling for demographic variables. These results were partially supported by the findings of previous studies showing that athletes’ sports injuries can be affected by LOC scores and that internal athletes tend to be tough and rational [14–17].

One of the interesting findings of this study was that significant gender differences were found in rational choice and tough factors. This study found that male athletes scored higher on rational choice and tough factors than female athletes. A previous study found that women’s emotions were more intense, lasted longer and expressed in more pronounced forms than men’s and men are more rational than women, can better control their emotions and are not easily impulsive [36]. Compared with male athletes, female athletes are more likely to adopt emotion-centered coping strategies when faced with risk, pain and injury, resulting in more anxiety [37]. This study also revealed that training years can predict the rational choice factor and the pressed factor. Thus, in future studies, we should focus on how to prevent sports injuries in athletes with longer and shorter training years.

Another important finding is that sports type affects RPIQ. Consistent with the findings of previous studies [27], this study found that athletes who participate in individual sports score higher on tough factor than those who participate in team sports, and athletes who participate in team sports show more resilience in terms of risk, pain and injury than athletes who participate in individual sports. However, some studies suggest that team athletes can rely on the strength and support of the team, while individual athletes can only rely on themselves, so the athletes in individual sports must make greater efforts on their own to achieve success [38].

Finally, the relationships between sport risk and the RPIQ factor also provide evidence for its usefulness in assessing athletes’ sport risk, pain and injury. Consistent with those of previous studies [25, 26], this study found that athletes who participated in low-risk sports scored higher on rational choice factor than those who participated in high-risk sports, although the difference was relatively small. With the increase in injury risk, athletes scored lower on the rational choice factor, indicating that athletes facing a higher degree of risk demonstrated a higher willingness to accept sports risk. Additionally, when faced with risk, pain and injury, athletes in high-risk sports were calmer and more rational than those in other sports [39].

## 5 Limitations and implications

There are limitations in this study. First, the relationships between the RPIQ-C factors and LOC might be underestimated due to self-report method was used. However, the main purpose of this study is to develop a reliable and validate instrument. As in previous studies [6, 7, 9], self-report method is the most suitable for assessing athlete’s risk, pain and injury. Another limitation is that SIAS and LOC were used to test the validity of the RPIQ-C in this study. In future studies, Anterior Cruciate Ligament-Return to Sport after Injury scale (ACL-RSI) and Injury Psychological Readiness to Return to Sport scale (I-PRRS) can be used to validate the RPIQ. A third limitation is that the sample in this study was relatively small, which might not represent the whole collegiate athletes populations in China. Future studies with a larger sample should be conducted to further assess its external validity.

The findings of this study have some practical implications. This study takes the first step in adapting the RPIQ to the Chinese language. As in previous studies [11], this study used collegiate students as participants. Future studies using professional athletes as participants are recommended to examine its reliability and validity. Second, this study found that LOC is negatively correlated with RPIQ-C factors, which suggests that interventions can be developed from the perspective of LOC. For athletes who are prone to injury in sports, changing their internal or external LOC might be of great importance.

## 6 Conclusion

The Chinese version of the RPIQ has sufficient psychometric properties and can be used as a reliable and effective tool for measuring attitudes of the risk, pain and injury of collegiate athletes. This study not only revealed significant correlations between the RPIQ-C factors, LOC and SIAS but also showed significant differences in the RPIQ-C factors by gender, risk of injury and type of sport. The findings of this study showed that the PRIQ-C is a reliable tool for understanding how Chinese athletes deal with risk, pain and injury and make decisions.

## Supporting information

S1 Fig The path diagram. (DOCX)

S1 File The Chinese version of RPIQ. (DOC)

S2 File The English version of RPIQ. (DOCX)
