# Supplementary figures and images for: Psychometric properties of the risk, pain, and injury questionnaire in Chinese collegiate athletes and its relationship with locus of control
Source: PLoS One. 2023 Jan 27;18(1):e0281011. doi: 10.1371/journal.pone.0281011 (PMC9882647; doi:10.1371/journal.pone.0281011)

**Appendix A**


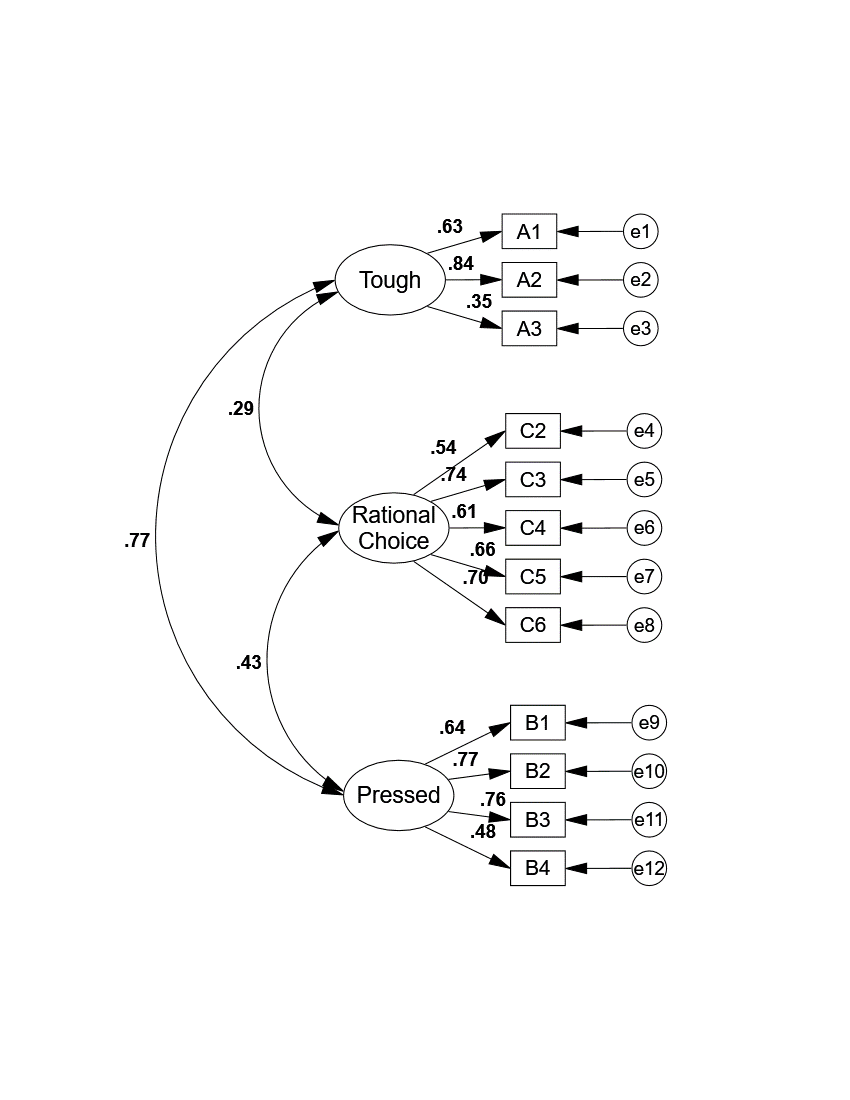
 **Fig 1.** Path diagram

Supplement: S1 Fig — (DOCX) [file pone.0281011.s001.docx]
