# Supplementary material for: Psychometric properties of the risk, pain, and injury questionnaire in Chinese collegiate athletes and its relationship with locus of control
Source: PLoS One. 2023 Jan 27;18(1):e0281011. doi: 10.1371/journal.pone.0281011 (PMC9882647; doi:10.1371/journal.pone.0281011)
Supplement: S1 File — (DOC) [file pone.0281011.s002.doc]

1. **运动员应该“坚持到底”今天的受伤或疼痛，而不是担心明天的影响。**

强烈同意 同意 不同意 强烈反对

1 2 3 4

1. **运动员应该忽略疼痛。**

强烈同意 同意 不同意 强烈反对

1 2 3 4

1. **在受伤和痛苦中比赛展示了性格和勇气。**

强烈同意 同意 不同意 强烈反对

1 2 3 4

1. **教练让运动员感到内疚，如果他们不希望伤害或痛苦。**

强烈同意 同意 不同意 强烈反对

1 2 3 4

1. **教练只关心他们的球员是健康的，能够比赛。**

强烈同意 同意 不同意 强烈反对

1 2 3 4

1. **教练说他们不希望运动员重伤,但是实际上如果他们是必要的，教练会把运动员推去比赛。**

强烈同意 同意 不同意 强烈反对

1 2 3 4

1. **教练们对带伤参赛的运动员印象深刻。**

强烈同意 同意 不同意 强烈反对

1 2 3 4

1. **那些忍受痛苦和遭受伤害的运动员应该得到我们的尊重。**

强烈同意 同意 不同意 强烈反对

1 2 3 4

1. **关心自己球队的运动员会试图带着伤痛参加比赛。**

强烈同意 同意 不同意 强烈反对

1 2 3 4

1. **每个运动员在比赛的时候都应该有受伤或疼痛的时候。**

强烈同意 同意 不同意 强烈反对

1 2 3 4

1. **只有运动员才明白带着伤痛打球是什么滋味。**

强烈同意 同意 不同意 强烈反对

1 2 3 4

1. **尽管有伤病和疼痛，运动员们将尽一切可能参加比赛。**

强烈同意 同意 不同意 强烈反对

1 2 3 4
