# Supplementary material for: Psychometric properties of the risk, pain, and injury questionnaire in Chinese collegiate athletes and its relationship with locus of control
Source: PLoS One. 2023 Jan 27;18(1):e0281011. doi: 10.1371/journal.pone.0281011 (PMC9882647; doi:10.1371/journal.pone.0281011)
Supplement: S2 File — (DOCX) [file pone.0281011.s003.docx]

**Tough**

1.No pain, no gain.

2.Athletes should “tough it out” with an injury or pain today and not worry about the effects tomorrow.

3.Athletes should ignore the pain.

4.Playing with injuries and pain demonstrates character and courage

**Pressed**

5.Coaches make athletes feel guilty if they do not want to play hurt or with pain

6.Coaches only care about their players who are healthy and able to play.

7.Coaches say they do not want athletes to play with serious injuries, but they actually push them to play if they are needed.

8.Coaches are impressed with athletes who play with injuries and pain.

**Rational Choice**

9.Athletes who endure pain and play hurt deserve our respect.

10.Athletes who care about their team will try to play with injuries and pain.

11.Every athlete should expect to have to play with an injury or pain sometime.

12.Only athletes understand what it is like to play with injuries and pain.

13.Athletes will do everything possible to play despite injuries and pain.
